# Inversion of the Chromosomal Region between Two Mating Type Loci Switches the Mating Type in *Hansenula polymorpha*

**DOI:** 10.1371/journal.pgen.1004796

**Published:** 2014-11-20

**Authors:** Hiromi Maekawa, Yoshinobu Kaneko

**Affiliations:** Yeast Genetic Resources Laboratory, Graduate School of Engineering, Osaka University, Osaka, Japan; Duke University Medical Center, United States of America

## Abstract

Yeast mating type is determined by the genotype at the mating type locus (*MAT*). In homothallic (self-fertile) Saccharomycotina such as *Saccharomyces cerevisiae* and *Kluveromyces lactis*, high-efficiency switching between **a** and α mating types enables mating. Two silent mating type cassettes, in addition to an active *MAT* locus, are essential components of the mating type switching mechanism. In this study, we investigated the structure and functions of mating type genes in *H. polymorpha* (also designated as *Ogataea polymorpha*). The *H. polymorpha* genome was found to harbor two *MAT* loci, *MAT1* and *MAT2*, that are ∼18 kb apart on the same chromosome. *MAT1*-encoded α1 specifies α cell identity, whereas none of the mating type genes were required for **a** identity and mating. *MAT1*-encoded α2 and *MAT2*-encoded **a**1 were, however, essential for meiosis. When present in the location next to *SLA2* and *SUI1* genes, *MAT1* or *MAT2* was transcriptionally active, while the other was repressed. An inversion of the *MAT* intervening region was induced by nutrient limitation, resulting in the swapping of the chromosomal locations of two *MAT* loci, and hence switching of mating type identity. Inversion-deficient mutants exhibited severe defects only in mating with each other, suggesting that this inversion is the mechanism of mating type switching and homothallism. This chromosomal inversion-based mechanism represents a novel form of mating type switching that requires only two *MAT* loci.

## Author Summary

The mating system of Saccharomycotina has evolved from the ancestral heterothallic system as seen in *Yarrowia lipolytica* to homothallism as seen in *Saccharomyces cerevisiae*. The acquisition of silent cassettes was an important step towards homothallism. However, some Saccharomycotina species that diverged from the common ancestor before the acquisition of silent cassettes are also homothallic, including *Hansenula polymorpha*. We investigated the structure and functions of the mating type locus (*MAT*) in *H. polymorpha,* and found two *MAT* loci, *MAT1* and *MAT2*. Although *MAT1* contains both **a** and *α* information, the results suggest that it functions as *MATα*. *MAT* **a** is represented by *MAT2*, which is located at a distance of 18 kb from *MAT1*. The functional repression of *MAT1* or *MAT2* was required to establish **a** or *α* mating type identity in individual cells. The chromosomal location of *MAT1* and *MAT2* was found to influence their transcriptional status, with only one locus maintained in an active state. An inversion of the *MAT* intervening region resulted in the switching of the two *MAT* loci and hence of mating type identity, which was required for homothallism. This chromosomal inversion-based mechanism represents a novel form of mating type switching that requires two *MAT* loci, of which only one is expressed.

## Introduction

**Figure 1:**
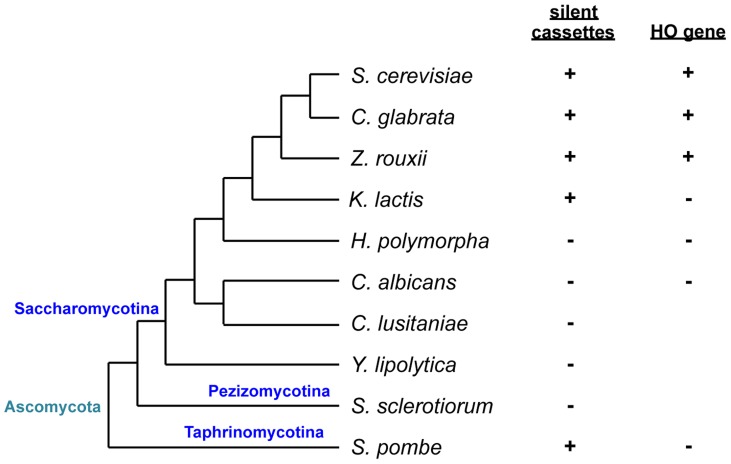
Schematic of phylogenetic relationships among yeast species and conservation of silent mating type cassettes and the *HO* gene. Information on silent cassettes and the *HO* gene is based on ref. 13. The tree is not drawn to scale.

Many yeast species have a sexual cycle as well as an asexual proliferation cycle. Sexual reproduction in yeast is initiated by the recognition of a mating partner and cell fusion, followed by nuclear fusion to form diploid cells that undergo meiosis and produce haploid progeny. In most ascomycetous yeast, cell-cell recognition only occurs between opposite mating types that are dictated by a single mating type locus, the *MAT* locus [1], which encodes transcriptional regulators that function in various combinations to regulate the expression of genes that confer a sexual identity to cells. Because mating type in Ascomycota is predominantly bipolar, there are two possible DNA sequences for the *MAT* locus, which are referred to as idiomorphs rather than alleles due to a lack of overall DNA sequence homology [2] (Figs. 1 and S1).

In *Saccharomyces cerevisiae*, haploid **a** or α cells are competent to mate with cells of the opposite mating type while diploid **a**/α cells are non-mating. The *S. cerevisiae MAT* locus carries one of two idiomorphs, *MAT* **a** or *MAT*α that encodes one or two proteins, **a**1 or α1 and α2, respectively. The α1 protein induces the expression of α-specific genes, while α2 represses **a**-specific genes. In contrast, the expression of **a**-specific genes does not require any of the *MAT* genes and occurs by default as long as α2 is absent [3]. This has resulted from the evolutionary loss of **a**2, another protein found in *MAT* **a** idiomorphs of several other Saccharomycotina species. In *Candida albicans* and *Candida lusitaniae*, **a**2 activates **a**-specific genes [4]. In diploid *S. cerevisiae* cells, α2 forms a complex with **a**1 to repress haploid-specific genes, which results in the loss of mating capability and gain of the ability to initiate meiosis [4].

Communication through mating pheromones is important in yeast mating [5]. In *S. cerevisiae*, pheromone and receptor genes are regulated by *MAT* [3]; the α-factor receptor, Ste2, and **a**-factor are expressed only in **a** cells and the **a**-factor receptor, Ste3, and α-factor only in α cells. Therefore, pheromone/receptor pairs can only be formed between **a** and α cells and mating can only occur between **a** and α cells. When bound by pheromone, both receptors activate the same downstream target molecules [6], and the signal is transmitted through the mitogen-associated protein kinase (MAPK) cascade—comprising Ste11, Ste7, and Fus3—to ultimately activate downstream effectors including the transcription factor Ste12, which then activates the expression of mating-specific genes [7]. The pheromone signal transduction pathway is highly conserved across fungi even beyond Ascomycota [8], [9].

Sexual reproduction can be heterothallic (cross-fertility), where mating occurs between individuals with compatible *MAT* idiomorphs, or else homothallic (self-fertility), where mating occurs within a population of the same strain. Two types of homothallism are known in yeast: in one, genetically identical cells mate with each other [10], while in the other, cells switch from one mating type to another, producing a cell population with two cell types that differ only in terms of *MAT* and are compatible to mate. The best characterized example of the latter is in *S. cerevisiae* which, in addition to the *MAT* locus, has silent copies of both idiomorphs at different locations on the same chromosome (*HML*α and *HMR* **a**) [3], [11], [12]. Cells switch mating type during the mitotic cycle and become sexually compatible with neighboring cells. Mating type switching is a gene conversion event that copies information from silent cassettes to the *MAT* locus and is initiated by a double-strand break generated by the HO endonuclease. While species related to *S. cerevisiae* such as *C. glabrata*, *Saccharomyces castellii*, and *Zygosaccharomyces rouxii* have silent mating type cassettes and the HO endonuclease gene, the silent cassette is absent in the most distantly related Saccharomycotina such as *C. albicans* or *Yarrowia lipolytica* [13] (Fig. 1). In more closely related yet still relatively distant yeasts such as *Kluveromyces lactis*, there are two silent cassettes but the HO endonuclease is absent. As in *S. cerevisiae*, mating type switching in *K. lactis* is mediated by mitotic gene conversion, but the initiating DNA lesion is evoked by a transposase homolog encoded by the *MATα* locus [14], [15].

*Hansenula polymorpha* is a more distantly related yeast used for genetic analyses, but the genetic and molecular details of its life cycle remain unknown. The species is predominantly haploid, but diploid cells can be isolated and maintained [16]. Because it is homothallic, haploid cells can mate with each other, followed by meiosis and sporulation under conditions of nutrient limitation. Diploid cells also efficiently undergo meiosis to form four ascospores [16], [17]. Mating type was suggested to be bipolar and the switching induced by nitrogen deprivation [16]. However, it was also claimed to be tetrapolar [16]. The genome sequence revealed the presence of the *MAT* locus but not silent cassettes or the *HO* gene. The *MAT* locus contains a unique combination of mating type genes—*α2*, *α1*, and **a** *1*—adjacent to each other on the same chromosome in that order and all in the same orientation [13]. However, it is not known how mating type is determined and whether and how the mating type switch occurs in this organism [13].

Here we report a functional analysis of mating type genes in *H. polymorpha*. Mutational analyses revealed that the previously reported *MAT* locus corresponds to *MAT*α, while *MAT* **a** is encoded by a second *MAT* locus located close to *MAT*α. Only one *MAT* locus was transcribed mitotically while the other was repressed. The chromosomal location determined which *MAT* was active. During mating, the chromosomal region between the two *MAT* loci became inverted, which resulted in the switching of the *MAT* locus that was expressed. Preventing the inversion severely perturbed the mating of cells with each other, suggesting that this is the major mechanism of homothallism in *H. polymorpha*.

## Results

### *H. polymorpha* has two mating type loci

**Figure 2:**
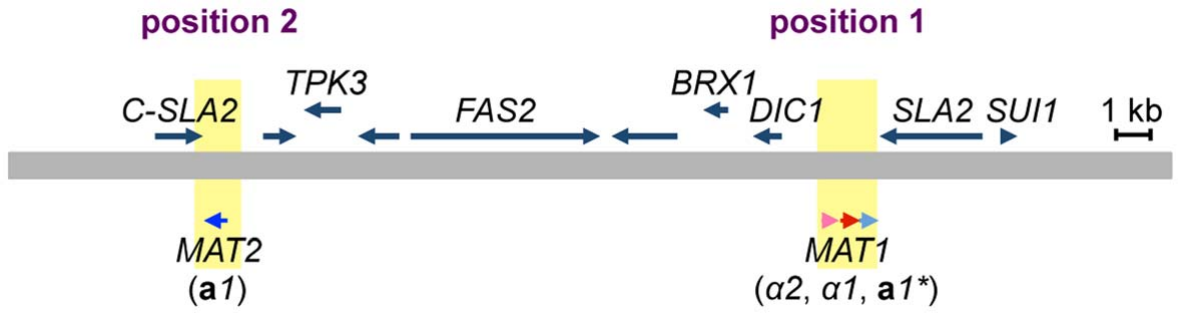
Two mating type loci in *H. polymorpha*. Schematic of the chromosomal region surrounding *MAT1* and *MAT2*. The chromosome is represented by a thick grey line; α*2*, α*1*, **a** 1* and **a** *1* genes are indicated by pink, red, and light and dark blue arrows, respectively. *MAT* loci are indicated by yellow. Predicted ORFs are indicated by blue arrows above the chromosome. Chromosomal positions proximal and distal to *SLA2* are marked as positions 1 and 2, respectively.

The *MAT* locus of *H. polymorpha* has been previously described as containing both *MAT* **a** and *MAT*α information on the same idiomorph, i.e., the α*2*, α*1*, and **a** *1* genes in that order [13] (Figs. 2, S1, and S2). In addition, the draft genome sequence of BY4329 (originally named SH4329) revealed a second **a** *1*-like gene, together with the C-terminal half of the *SLA2* gene, about 18 kb upstream of α*2* in the opposite orientation (Fig. 2). The predicted amino acid sequences of the two **a**1-like proteins were identical except for the N-terminal 24 amino acids (Fig. S3). Amino acid similarity to *S. cerevisiae* **a**1 was detected only within the identical sequences (Fig. S3). A similar genome structure was reported for the closely related yeast *Ogataea parapolymorpha* DL-1 [18]. Hereafter, the **a** *1* gene of the previously reported *MAT* locus and the second **a** *1*-like open reading frame (ORF) are referred to as **a** 1* and **a** *1* genes, respectively, and mating type loci containing them are referred to as the *MAT1* and *MAT2* loci, respectively, since both are expressed and function in the sexual cycle (Fig. 2, see below).

### Mating partner recognition requires two pheromone receptor homologs

**Figure 3:**
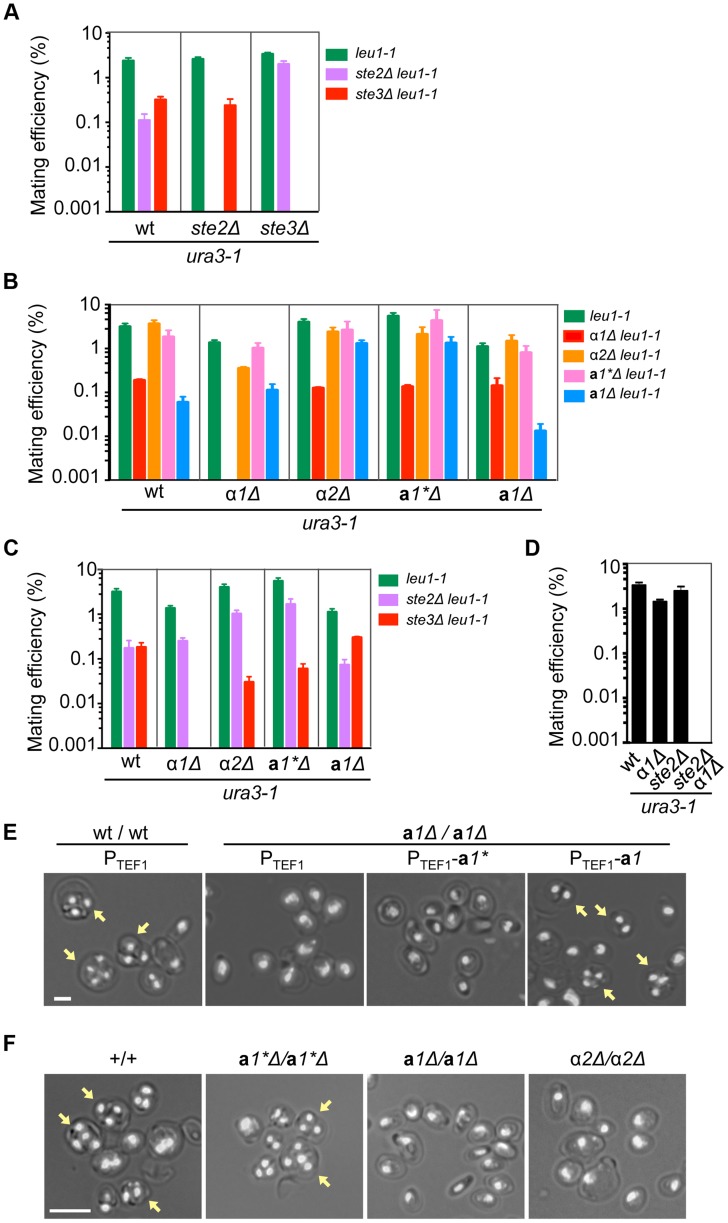
Functions of mating type genes in sexual development. (A) Two mating pheromone receptors are required for mating. Wild-type, *ste2*Δ and *ste3*Δ *H. polymorpha* strains of *ura3-1* (BY4330, HPH555, and HPH582 respectively) and *leu1-1* (HPH22, HPH553, and HPH581 respectively) genotypes were combined on MEMA mating medium and incubated at 30°C. After 24 h, cells were spread on SD plates to select for Leu+Ura+ diploids. Colony number was counted after 2 days at 37°C. Shown is the average of three independent matings. Error bars indicate SD. (B) Mating assay of wild-type, α*1*Δ, α*2*Δ, **a** 1*Δ, and **a** *1*Δ strains. Wild-type (HPH22 and BY4330), α*1*Δ(HPH546 and HPH548), α*2*Δ (HPH329 and HPH331), **a** 1*Δ (HPH517 and HPH521), and **a** *1*Δ (HPH675 and HPH678) strains were treated as described in (A). Shown is the average of three independent matings. Error bars indicate SD. (C) Mating assays of wild-type (HPH22 and BY4330), *α1*Δ (HPH546 and HPH548), *α2*Δ (HPH329 and HPH331), **a** 1*Δ (HPH517 and HPH521), and **a** *1*Δ (HPH675 and HPH678) strains with *ste2*Δ (HPH553 and HPH555) and *ste3*Δ (HPH581 and HPH582) strains. Cells were treated as described in (A). Shown is the average of three independent matings. Note that *ste2*Δ and *ste3*Δ strains behave as heterothallic α or **a** strains, respectively. Error bars indicate SD. (D) Mating assay for the *α1*Δ *ste2*Δ strain. Wild-type (BY4330), *α1* (HPH548), *ste2*Δ (HPH555), and *α1*Δ *ste2*Δ (HPH642) strains of the *ura3-1* genotype were combined with a wild-type strain of the *leu1-1* (HPH22) genotype as described in (A). Shown is the average of three independent matings. Error bars indicate SD. (E) **a** 1* and **a** *1* are functionally distinct. Logarithmically growing wild-type diploid (HPH723) and **a** *1*Δ homozygous diploid (**a** *1*Δ*/* **a** *1*Δ; HPH724) cells carrying the indicated plasmid were spotted on MEMA plates and incubated at 30°C for 24 h. Plasmids used were pHM850 (P_TEF1_), pHM848 (P_TEF1_-**a** 1*), and pHM849 (P_TEF1_-**a** *1*). Shown are merged brightfield and DAPI epifluorescence images. Yellow arrows indicate spores. Bar, 2 µm. (F) Functions of **a** *1* and *α2* are essential for meiosis and sporulation. Cells were prepared as described in (E). Shown are merged brightfield and DAPI epifluorescence images. Yellow arrows indicate spores. Bar, 5 µm.

To elucidate the molecular mechanism of homothallism in *H. polymorpha*, the contribution of each mating type gene to the sexual cycle, i.e. mating and meiosis/sporulation, was investigated. To this end, we first sought cells that behaved like heterothallic **a** and α cell type strains in mating and meiosis. *H. polymorpha* genome sequences contain ORFs homologous to *S. cerevisiae STE2* and *STE3* genes encoding α- and **a**-factor receptors, respectively [19], [20]. *Ste2*Δ and *ste3*Δ strains were generated that were expected to behave as heterothallic α and **a** cell types, respectively, and therefore unable to self-mate, while cross-mating was possible. The mating capability of the strains was determined by a semi-quantitative mating assay. When *H. polymorpha* mate successfully, the resulting diploid cells (zygotes) immediately undergo meiosis and sporulation, provided that nutrients remain limited. However, if nutrients are supplied after mating and before the commitment to meiosis, cells return to the proliferative state as diploids. We took advantage of this life cycle to evaluate mating efficiency based on the number of diploid colonies formed after return to growth. Although *Ste2*Δ and *ste3*Δ cells produced comparable numbers of diploids when crossed with wild-type cells or with each other, no diploids were observed from the *Ste2*Δ × *Ste2*Δ and *ste3*Δ × *ste3*Δ crosses (Fig. 3A). These results suggest that mating is bipolar in the homothallic laboratory strain derived from NCYC495, and that *Ste2*Δ and *ste3*Δ cells can undergo mating only as α and **a** cells, respectively.

### α*1* is required for mating, whereas α*2* and a*1* have roles in meiosis and sporulation

Genetic and phenotypic analyses of mating type gene deletion mutants were carried out to determine the functional roles of the **a**1, **a**1*, α1, and α2 transcription factors. Mating capability was evaluated by the semi-quantitative mating assay and meiosis/sporulation was determined by microscopy.

Although mating efficiency was generally low (<∼2% after 24 h) and varied widely among strains, deleting the α*1* gene nearly abolished mating with cells of the same genotype (i.e., α*1*Δ × *α1*Δ; Fig. 3B). There were no signs of mating such as zygotes and altered cell morphology (i.e., mating projections) detected by microscopy. In contrast, **a** 1*Δ, **a** *1*Δ, and α*2*Δ cells exhibited normal mating behavior and produced homozygous diploids in crosses with cells of the same genotype (i.e., α*2*Δ × *α2*Δ, **a** 1*Δ × **a** 1*Δ, and **a** *1*Δ × **a** *1*Δ; Fig. 3B), although the efficiency was lower for the **a** *1*Δ × **a** *1*Δ cross than for other combinations. Interestingly, α*1*Δ cells were able to mate with *Ste2*Δ cells, but did not produce diploids when mated with *ste3*Δ (Figs. 3C and S4A). Furthermore, α*1*Δ *ste2*Δ cells did not mate with wild-type cells (Figs. 3D and S4B). In contrast, *Ste2*Δ cells could mate with all mutants of mating type genes (Figs. 3C and S4A). Thus, *α1* but not *α2* determines the *α* cell identity and is indispensable for mating. The **a** cell identity may be established by default, as is the case in *S. cerevisiae*, because neither the **a** 1* nor the **a** *1* gene was essential for mating. Support for this conjecture comes from the observation that constitutive expression of the *α1* gene strongly inhibited mating with *Ste2*Δ (*α* cell-like) but not with *ste3*Δ (**a** cell-like) (Fig. S5).

Although **a** *1* and α*2* were not required for mating, homozygous diploids of **a** *1*Δ or α*2*Δ (**a** *1*Δ*/* **a** *1*Δ and α*2*Δ*/*α*2*Δ) did not undergo meiosis nor did they produce spores (Fig. 3E, F). In contrast, **a** 1*Δ*/* **a** 1*Δ diploid cells exhibited normal meiosis/sporulation (Fig. 3F). Since the amino acid sequences of **a** 1* and **a** *1* are identical except for the N-terminal 24 amino acids (Fig. S3), the possibility of functional redundancy was examined. Meiotic deficiency of **a** *1*Δ*/* **a** *1*Δ diploid cells was not suppressed by expressing the **a** 1* gene from the constitutive *HpTEF1* promoter[21], while **a** *1* expression restored normal meiosis and sporulation (Fig. 3E), suggesting that the two genes have distinct functions. Thus, α*1* has an essential role in mating while **a** *1* and α*2* are indispensable for meiosis and sporulation, in a manner analogous to *S. cerevisiae*. Because **a** 1* was not involved in sexual differentiation, we concluded that *MAT1* and *MAT2* represent α and **a** mating types, respectively.

### Inversion of the region between *MAT1* and *MAT2* alters *MAT* gene expression

**Figure 4:**
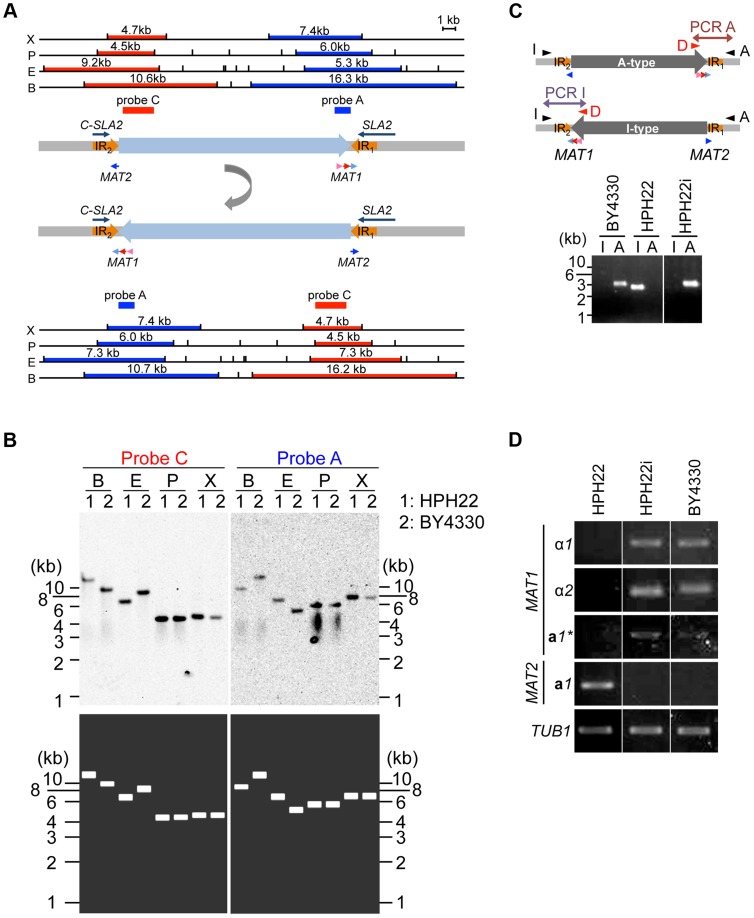
Inversion of the *MAT* intervening region alters the expression status of mating type genes. (A) Schematics of the chromosomal region surrounding *MAT1* and *MAT2*. α*1*, α*2*, **a** 1*, and **a** *1* genes are indicated by pink, red, and light and dark blue arrows, respectively. IR regions are shown as thick orange arrows. DNA fragments used as probes for Southern blot analysis in (B) are shown as dark blue (probe A) or red (probe C) bars. The upper schematic shows the draft genome sequence. The lower schematic shows the predicted DNA sequences after the inversion between IR regions. Upper and lower panels show restriction enzyme sites deduced from the DNA sequences and the size of the DNA fragment hybridized by each probe. X, *Xho*I; P, *Pst*I; E, *Eco*RI; B, *Bam*HI. (B) Two types of chromosome configuration in different wild-type strains. Genomic DNA of HPH22 (indicated as 1) and BY4330 (indicated as 2) were prepared from logarithmically growing cells in YPDS medium and analyzed by Southern blotting using probes A and C (upper panel). The lower panel shows the results predicted from Upper (U) and Lower (L) schematics in (A). (C) PCR amplification of the I- or A-type *MAT1* locus (reaction I with Primer_I/Primer_D or reaction A with Primer_A/Primer_D). The presence of the I product and absence of the A product for HPH22 indicates that the chromosome is in the I-type orientation. HPH22i and BY4330 have an A-type chromosome. (D) Mating type genes are transcriptionally active at position 1 and repressed at position 2. The expression of α*1*, α*2*, **a** 1*, and **a** *1* genes was examined by RT-PCR. RNA samples were prepared from logarithmically growing wild-type cells in YPDS medium at 30°C (HPH22, HPH22i, and BY4330). HPH22i is a clone isolated from HPH22 (Fig. S4B; see text).

The sequences 2049 bp downstream of *MAT1* and upstream of *MAT2* (referred to as IR_1_ and IR_2_, respectively) are identical (Fig. 4A). Since PCR amplification of the region spanning IR_1_ or IR_2_ often yields ambiguous results (Fig. S6A), Southern blot analysis was used to verify genome sequences surrounding the two *MAT* loci. Genomic DNA was prepared from the laboratory wild-type strains HPH22 (derived from BY4329) and BY4330 (originally named SH4330), and DNA fragments encompassing *MAT1* and in close proximity to *MAT2* were used as probes A and C, respectively. Results for BY4330 matched our draft genome sequences, but for HPH22, a match was observed only if the sequences between IR_1_ and IR_2_were presumed to be inverted (Fig. 4A, B). To investigate whether the orientation of this region differed in the two strains, two PCR reactions were carried out in which only one orientation was amplified (Fig. 4C). A PCR product was observed for only one reaction using BY4330 and the other reaction using HPH22 (Fig. 4C), indicating that there are two distinct genomic structures surrounding the *MAT* loci.

The conservation of gene order flanking the *MAT1* locus has been previously noted [13]. The presence of the *SLA2* and *SUI1* genes downstream of the *MAT* locus is conserved among yeast species distantly related to *S. cerevisiae* such as *Saccharomyces kluyveri*, *K. lactis*, and *Y. lipolytica* [13](Fig. S1). Furthermore, the *DIC1* gene is located on the other side of *MAT* in *S. kluyveri*. Based on this conserved gene order, BY4330 likely reflects the ancestral type. Therefore, the BY4330 and HPH22 types are hereafter referred to as ancestral (A)- and inverted (I)-type, respectively (Fig 4C). In addition, the ancestral chromosomal location of *MAT* and the 2nd location are referred to as positions 1 and 2, respectively (Fig. 2).

After an additional 5–10 amplification cycles, specific products often appeared in both PCR reactions (Fig. S6A). Furthermore, although most single colonies isolated from HPH22 maintained the I-type orientation, some isolates such as HPH22i became A-type (Fig. 4C). Further isolates obtained from HPH22i (15 out of 16) remained as A-type (Fig. S6B). These results suggest that the switch between I- and A-types can occur in mitotically growing cells, albeit at a low frequency. Moreover, once inversion takes place, the new orientation is stably maintained.

Given that information for both *MAT* **a** and *MAT*α co-exist in a single cell but cells are nonetheless competent for mating, the possibility that the transcription of mating type genes are differentially regulated was investigated. Reverse transcriptase PCR (RT-PCR) analysis of mitotically growing HPH22 cells revealed that the **a** *1* gene but not genes at the *MAT1* locus (α*1*, α*2*, and **a** 1*) are expressed (Fig. 4D). In contrast, three genes at *MAT1* were expressed while the **a** *1* gene at *MAT2* was repressed in BY4330 cells (Fig. 4D). The differences in *MAT* gene expression patterns were not due to different genetic backgrounds, but were instead dependent on the chromosomal arrangement surrounding *MAT* loci (A- or I- type), because HPH22i exhibited the same type of expression as BY4330 (Fig. 4D). This suggests that both *MAT1* and *MAT2* are transcriptionally active at position 1, but are repressed at position 2.

Although α*1*, α*2*, **a** 1* RNA was detected by RT-PCR, it is unclear whether these are transcribed individually. The α*2* and α*1* ORFs are separated only by a 5-bp gap, while a 19-bp overlap exists between α*1* and **a** 1* (Fig. S2). Indeed, we detected RNA species that carry both α*1* and **a** 1* ORFs (Fig. S7).

### Mating type switching is induced by nutrient starvation

**Figure 5:**
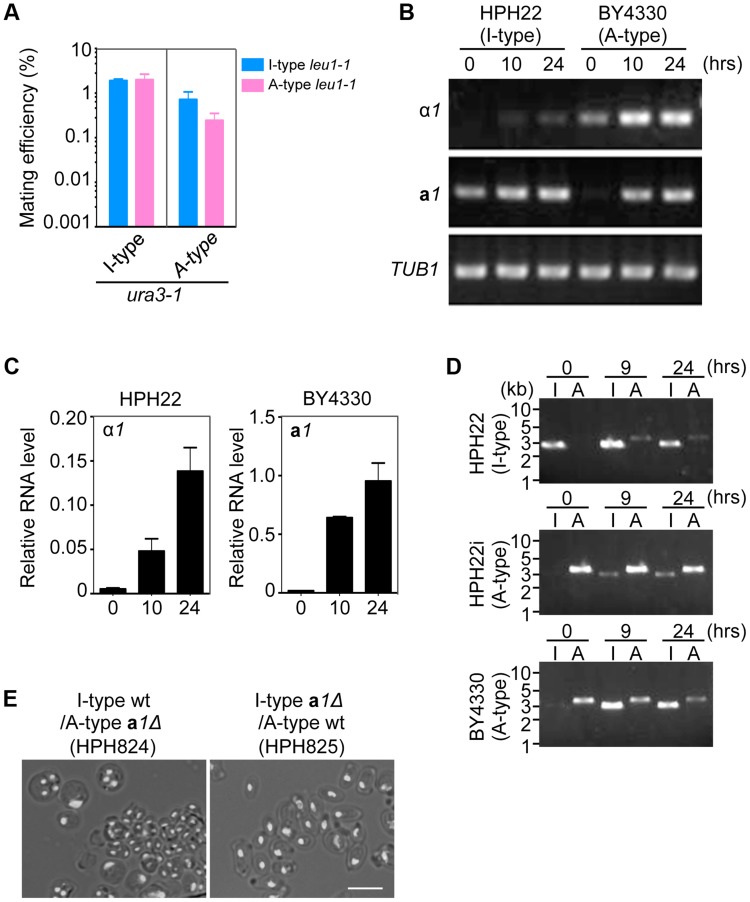
Inversion of the *MAT* intervening region is induced during mating. (A) Mating assay between I (HPH22 and HPH719)- and A (HPH22i and BY4330)-type strains. Cells were treated as described in Fig. 3A. Shown is the average of three independent matings. Error bars indicate SD. (B) RT-PCR analysis of α*1* and **a** *1* genes. RNA samples were prepared from I (HPH22)- or A (BY4330)-type wild-type cells incubated on MEMA medium for the indicated times. Primers used for PCR are listed in Table S2. (C) Quantitative digital PCR analysis of α*1* and **a** *1* genes. RNA samples in (B) were subjected to digital PCR analysis. α*1* and **a** *1* RNA levels were normalized to that of *ACT1* RNA. Shown are the averages of two independent PCR reactions. Error bars indicate SD. (D) PCR amplification of the I- or A-type *MAT1* locus. PCR reactions are as described in Fig. 4C. Genomic DNA samples were prepared from three wild-type strains (HPH22, HPH22i, and BY4330) after incubation on MEMA for the indicated times. The appearance of the I product in the reaction with BY4330 (A-type) after 9 and 24 h indicates a switch to the I-type in a subset of the population. (E) Meiosis in **a** *1*Δ/+ heterozygous diploid cells. **a** *1*Δ (I-type)*/+* (A-type) is defective in meiosis. Cells were prepared as described in Fig. 4E. Shown are merged brightfield and DAPI epifluorescence images. Bar, 5 µm.

Because *MAT1* and *MAT2* represent α and **a** mating types, respectively, and the mating type identity of cells was determined by the chromosomal arrangement of *MAT* loci (A- or I- type), it was predicted that mating efficiency would be higher when the A- and I-types were mixed than for either type alone. However, combining A- or I- types had no effect on mating efficiency (Fig. 5A). This might suggest that the mating type identity of cells frequently switches under mating conditions regardless of the mating type during mitotic growth.

Next, the expression of mating type genes under mating conditions was investigated. In addition to α*1*, the expression of **a** *1* was induced in the A-type strain BY4330 after a 10-h incubation on the mating medium, MEMA (Fig. 5B, C). Similarly, the α*1* transcript was upregulated in the I-type strain HPH22 during mating although the induction was weaker than that of **a** *1* in BY4330 (Fig. 5B, C). Thus, all mating type genes were expressed during mating, providing an explanation for the self-mating observed in all examined strains.

The transcriptional activation of the *MAT* locus at position 2 after transfer to the mating medium could be due to de-repression of the repressed *MAT* locus. Alternatively, the inversion of the *MAT* intervening region could bring the repressed *MAT* locus to a transcriptionally active location. In the latter instance, the inversion would be frequently observed under starvation conditions. To investigate this possibility, logarithmically growing HPH22, HPH22i, and BY4330 cells were transferred to mating medium and chromosome orientation was evaluated by PCR (Fig. 5D). In all three strains, the inverted orientation became apparent under starvation conditions. The inversion might be more efficient in BY4330 than in HPH22, which could explain the stronger induction of **a** *1* mRNA in BY4330 as compared to α*1* mRNA in HPH22 under these conditions (Fig. 5B, C). These results support the notion that the inversion of the *MAT* intervening region is responsible for transcriptional induction.

The above results do not exclude the possibility that de-repression of the *MAT* locus at position 2 contributes to mating. In this scenario, the resulting diploids would harbor two chromosomes of the same type; therefore, chromosome types in diploid clones were examined. All 146 diploids isolated from all combinations of crosses had one I- and one A-type chromosome (Table 1). Thus, it is unlikely that transcription from the *MAT* locus at position 2 contributes significantly to mating. To further confirm the transcriptional status at position 2, meiosis was examined in diploid cells heterozygous for **a** *1*Δ (**a** *1Δ/+*). Diploid cells carrying the **a** *1*Δ allele on an A-type chromosome would be expected to express meiotically indispensable **a**1 protein from the **a** *1* gene at position 1 on an I-type chromosome. As predicted, such diploid cells underwent efficient meiosis and sporulation (HPH824; Fig. 5E). On the contrary, diploid cells carrying **a** *1*Δ on an I-type chromosome would only be capable of meiosis if the **a** *1* gene at position 2 were expressed. Indeed, meiosis was severely perturbed in these cells (HPH825; Fig. 5E). These results suggest that mating type genes at position 2 are not transcribed or else activated at subthreshold levels that are insufficient to induce meiosis.

**Table 1:** Chromosome type in diploid isolates.

	Chromosome types in diploid (%)			
cross	I/I	I/A	A/A	n
I-type x I-type	0	100	0	64
I-type x A-type	0	100	0	57
A-type x A-type	0	100	0	25

Diploid clones isolated from crosses of I- and A-type wild-type strains. I- or A-type was determined as described in Fig. 4C. *H. polymorpha* strains used were HPH22, HPH22i, HPH462, HPH468, HPH719, HPH720, and BY4330.

### Homothallism in *H. polymorpha* relies on inversion

**Figure 6:**
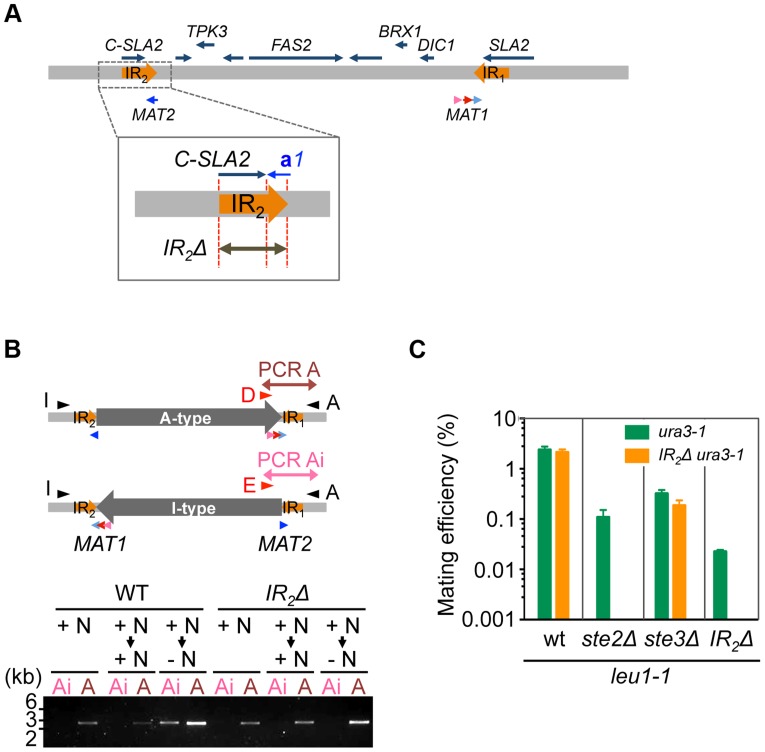
Inversion of the *MAT* intervening region is essential for homothallism. (A) Schematic of the strategy for IR_2_ deletion on the A-type chromosome. (B) *IR_2_*Δ cells are defective for the inversion. Logarithmically growing wild-type (HPH22i) and *IR_2_*Δ (HPH833) cells in YPDS medium (+N, nutrient plus) were transferred to YPDS (+N → +N) or MEMA (+N → −N, nutrient minus) and incubated for 20 h. Genomic DNA samples were prepared and inversion was detected by two PCR reactions, A and Ai, using the primer sets Primer_D/Primer_A and Primer_E/Primer_A, respectively. (C) Cells of the A-type strain carrying the *IR_2_*Δ allele are incapable of mating with each other and with *ste2*Δ. Wild-type (HPH22 and SH4330), *ste2*Δ (HPH553), *ste3*Δ (HPH581), and *IR_2_*Δ (HPH833 and HPH835) cells were treated as described in Fig. 3A. Shown is the average of three independent matings. Error bars indicate SD.

The aforementioned data strongly suggests that the A- and I- inversion types correspond to α and **a** mating types, respectively, and that inversion of the *MAT* intervening region is the major mechanism of mating type switching. To test this model, inversion-deficient mutants were generated. Because IR sequences likely play an important role in the inversion, an IR_2_ deletion was introduced (Fig. 6A), which abolished inversion in A-type (α) cells after transfer to the mating medium (Figs. 6B and S8). Mating with each other and with *Ste2*Δ cells was almost abolished in these cells, while mating with *ste3*Δ was unaffected (Fig. 6C). These results suggest that inversion after nutrient starvation is necessary for mating type switching, and is responsible for homothallism in *H. polymorpha*.

## Discussion

The *MAT* locus of *H. polymorpha* contains information for both *MAT*α and *MAT* **a**, which has been proposed as an explanation for homothallism in this haploid species. However, the presence of two different mating types and the mechanisms through which sexual compatibility is established have not been previously examined. Here we report the complete set of mating type genes and their roles during sexual development. The results suggest that mating type identity is determined by which of the two *MAT* loci are present at the actively expressed locus, and that homothallism results from the inversion of the intervening chromosomal region to result in mating-type switching.

### Sexual compatibility in *H. polymorpha*

**Figure 7:**
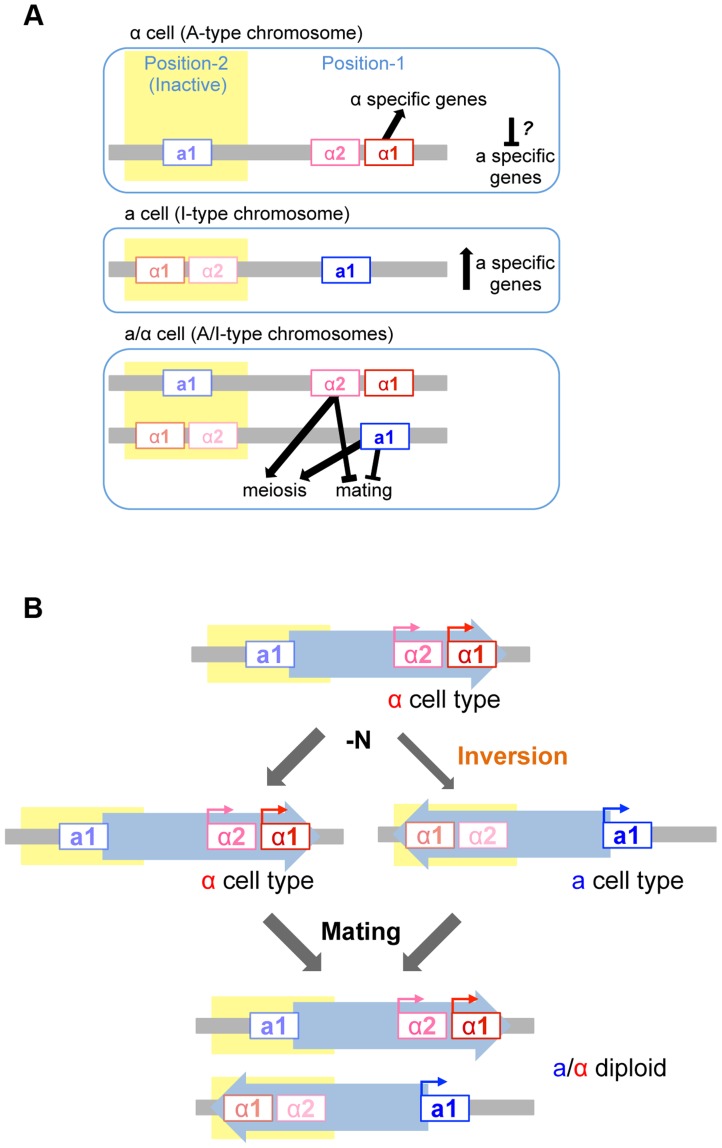
Model of mating type regulation in *H. polymorpha*. (A) Function of mating type genes in establishing mating type identity. (B) Model of homothallism in *H. polymorpha*.

Mutational analyses revealed that mating and meiosis are regulated by the distinct functions of four mating type gene products in *H. polymorpha* (Fig. 7A). The activation of haploid-specific genes is likely to be regulated in a manner similar to what is presumed for mating type genes of *S. cerevisiae*, although genes that are expressed specifically in **a**-, α-, or haploid cells have not yet been identified in *H. polymorpha*. In *S. cerevisiae* haploid cells, α*1* is essential for α-specific gene expression, while **a**-specific genes are expressed by default and do not require any mating type genes. Thus, in this species, **a** cell identity is established by default unless α2 represses **a**-specific genes [1]. Establishment of α identity requires the activation of α-specific genes by α1 in addition to the repression of **a**-specific genes. However, in *H. polymorpha*, α2 is not involved in the repression of **a**-specific genes, and it is therefore unclear how these are repressed in α cells. It is currently unknown whether the repression of **a**-specific genes is necessary in α cells. One possibility is that α1 contributes to this repression, as was suggested in *C. lusitaniae* [4]. Alternatively, there may be no mechanism to repress **a**-specific genes, in which case the intrinsic noise of gene expression may create different populations that express variable levels of α1 and **a**-specific genes. Cells may therefore exhibit *α* identity during a time window during which α1 level is high and **a**-specific gene expression is low.

### Repression of the *MAT* locus

The *MAT* loci in *H. polymorpha*—*MAT1* and *MAT2*—are transcriptionally active at position 1, the ancestral location. However, their transcription became repressed at position 2 after the inversion of the *MAT* intervening region. The promoter sequences of mating type genes were not responsible for the repression, since sequences upstream of mating type genes were unaltered; instead, the orientation of mating type genes and others within the *MAT* intervening region was reversed. However, this was unlikely to repress transcription. Indeed, the expression of the *FAS2* gene located in the middle of the *MAT* intervening region was independent of A- or I-type arrangement and nutrient starvation (Fig. S9) [22]. The most plausible explanation is that position 2 is in a silent configuration. This is supported by the fact that there are no ORFs in the >12 kb region next to IR_2_ distal to position 1, except for one encoding the polyprotein-like protein of the Ty/Copia retrotransposon. It may also explain why IR_2_ deletion could not be rescued by a DNA fragment containing a selection marker of similar size. Whether the repression at position 2 depends on heterochromatin structure is unknown. However, it is worth noting that, like other Saccharomycotina, there are no Heterochromatin Protein 1 family members in *H. polymorpha*, nor any clear homologs of *S. cerevisiae* trans-acting silencing proteins such as Sir1, Sir3, and Sir4 [23]-[26], although a histone deacetylase homologous to *S. cerevisiae* Sir2/Hst1 is present. *H. polymorpha* may have a silencing mechanism in which the Sir2 homolog plays a critical role and the Orc1 homolog possesses a Sir3-like silencing function as in *K. lactis* [27]. A Sir4 homolog may be too diverse to detect based on amino acid sequence similarity [28].

### Transcriptional circuit of sexual programs in *H. polymorpha*

Mating and meiosis are distinct programs in *S. cerevisiae* but are integrated in *H. polymorpha*. A similar sexual cycle occurs in the Saccharomycotina species *C. lusitaniae* and the distantly related Taphrinomycotina species *S. pombe* [4]. A recent study on the mechanism of sexual programs in *C. lusitaniae* has revealed the co-regulation of mating- and meiosis-specific gene expression programs [29]. In *S. cerevisiae*, the pheromone-associated transcription factors Ste12 and Ime2 are specifically involved in mating and meiosis, respectively. In contrast, *C. lusitaniae* Ste12 and Ime2 orthologs are required for efficient progression through both mating and meiosis. The absence of α2, which prevented expression of haploid-specific genes, including MAPK genes, was proposed to facilitate MAPK signaling and confer a meiotic role to Ste12. The coupling of mating and meiosis may have evolved to ensure the return of diploids to the haploid state to satisfy the preference for haploidy [29]. The same argument could be applied to the sexual cycle of the predominantly haploid *H. polymorpha*. Nonetheless, there are species differences in the expression of components essential for sexual regulation. In both *C. lusitaniae* and *S. pombe*, the transcription of genes encoding pheromone receptors and pheromone-associated transcription factors is induced during mating, but these are constitutively expressed in mitotically growing *H. polymorpha* cells (Fig. S10) [4], [30]–[32]. The evolution of this constitutive expression and the mechanisms involved in its regulation will be a focus of future studies.

### Mechanism of homothallism

Mating type switching has been best studied in *S. cerevisiae*, *K. lactis*, and *S. pombe* [14], [15], [33]. These species all harbor silent cassettes in their genomes and their switching events are mitotic recombination-dependent, although the molecular details differ. Homothallism in *H. polymorpha* involves two independent regulatory processes: transcriptional repression of one *MAT* locus, and inversion of the chromosomal region between the two *MAT* loci—*MAT1* and *MAT2*—that reside ∼18 kb apart on the same chromosome and are idiomorphs for the α and **a** mating types, respectively (Fig. 7B). Both *MAT* loci are active in the ancestral chromosomal position (position 1) while the other locus (at position 2) is repressed. The inversion of the *MAT* intervening region is induced under mating conditions, resulting in a chromosome that harbors the formerly repressed mating type genes at the active location and establishes the opposite mating type identity. Because this system differs from those of *S. cerevisiae* and *K. lactis*, it is likely to have evolved independently after *H. polymorpha* branched out from Saccharomycetaceae. Interestingly, the organization of *MAT1* is similar to that observed in homothallic Pezizomycotina such as *Sclerotiniasclerotiorum* and some Cocliobolus species [34], [35]. In the former, the inversion of part of the *MAT* locus leads to mating type switching [36]. Thus, the fusion of two *MAT* idiomorphs of the heterothallic ancestor followed by the acquisition of mitotic recombination to differentiate the two transcriptional profiles likely occurred multiple times during fungal evolution. In *H. polymorpha*, the insertion of a retrotransposon found in close proximity to position 2 may have caused the duplication of the IR region that contains most of the **a** 1*/**a** *1* ORF and then initiated an inversion event between the two IR regions. The two *MAT* loci in *H. polymorpha* may therefore represent an intermediate state preceding the acquisition of a set of silent cassettes. Comparative studies in other fungal species would be required to evaluate this possibility.

The molecular mechanism underlying the inversion in *H. polymorpha* is currently unknown. Well-studied examples of inversion-dependent phenotypic switching include phase variation systems in bacteria, such as Type 1 fimbrial phase variation in *Escherichia coli* and flagellar phase variation in *Salmonella enterica*, where the inverting regions contain a promoter for adjacent genes that determine the phenotype, with inversion therefore resulting in transcriptional on/off switching. In these cases, nonhomologous, site-specific serine or tyrosine families of recombinases act on inverted repeats, which leads to the inversion of the intervening sequence [37], [38]. In *S. cerevisiae*, the site-specific FLP tyrosine recombinase is an essential part of the 2-µm plasmid amplification system [39]. It will be interesting to determine whether inversion in *H. polymorpha* depends on site-specific recombination. However, there were no serine or tyrosine recombinases in the genome. Given that long homologous sequences (>2 kb) are in inverted orientations (IR_1_ and IR_2_), homologous recombination between IR regions is another possible mechanism leading to inversion of the *MAT* intervening region.

Although inversion is observed at low frequency during mitotic growth, it is strongly induced upon nutrient starvation in *H. polymorpha*. It is interesting that mating type switching is induced and mating is initiated in response to harsh environmental conditions such as nutritional starvation in *K. lactis* [40]. Elucidating the molecular mechanisms and regulation of mating type switching in *H. polymorpha* can provide deeper insight into how mating type switching evolved.

## Materials and Methods

### Yeast strains and plasmids

Strains and plasmids used in this study are listed in Table S1. Unless otherwise indicated, yeast strains were derived from NCYC495 [41] and were generated by PCR-based methods [42], [43]. Gene deletion alleles were generated in *ku80Δ* or *ku70Δ* cells and then crossed with either HPH22 or BY4330 to obtain *KU80^+^* or *KU70^+^* cells carrying the deletion allele. Primers used to amplify cassettes are listed in Table S2. *H. polymorpha* cells were transformed by electroporation [44]. pSC6cen103a is a newly developed plasmid stably maintained in *H. polymorpha*, the construction of which will be described elsewhere. The *HpURA3* DNA fragment containing 800 bp upstream and 500 bp downstream sequences was amplified by PCR and inserted into *Aat*II/*Sac*I sites in pRS305 to generate pHM821. The 500-bp sequences up- and downstream of the *HpTEF1* ORF were used as the *HpTEF1* promoter and terminator, respectively [21].

### Yeast growth conditions and general methods

Yeast strains were grown in yeast extract, peptone, and dextrose medium containing 200 mg/l adenine, leucine, and uracil (YPDS) [45]. Diploid cells were grown in synthetic/defined (SD) medium supplemented with appropriate amino acids and nucleotides. Cells were grown at 30°C unless otherwise indicated. Mating and meiosis were induced on 2.5% maltose and 0.5% malt extract medium (MEMA) plates at 30°C.

### Microscopy

Yeast cells were fixed with 70% ethanol, washed with phosphate-buffered saline (PBS), and incubated in PBS containing 1 µg/ml 4′6,-diamidino-2-phenylindole (DAPI) to visualize DNA. Images were acquired using the DeltaVision Personal system (Applied Precision, Issaquah, WA, USA). A Z series in 0.4-µm steps was acquired for DAPI images, and ImageJ (National Institutes of Health, Bethesda, MD, USA) was used to generate projected images. Adobe Photoshop (Adobe Systems, Inc., San Jose, CA, USA) was used to process and produce merged images.

### Genome sequencing and determination of A- or I-type

The BY4329 genome was sequenced using a Genome Sequencer FLX System (Roche Diagnostics, Basel, Switzerland) and Genome Analyzer GAIIx (Illumina Inc., San Diego, CA, USA). The paired-end library for the former was prepared according to the Paired-End Library Preparation Method Manual −20 kb and 8 kb Span (Roche Diagnostics), and a genome library for the latter was prepared with a TruSeq DNA Sample Preparation v2 Kit (Illumina Inc.) according to the manufacturer's protocol. All reads were assembled into contigs and then ordered into scaffolds using GS De Novo Assembler version 2.6 (Roche Diagnostics). The draft sequence data is submitted to DNA Data Bank of Japan (DDBJ) and its BioProject ID is PRJDB3035.

To determine the orientation of the region between IR_1_ and IR_2_, PCR primers specific for the sequence to the left of IR_2_ (Primer_I), the intervening region (Primer_D and Primer_E), and the sequence to the right of IR_1_ (Primer_A) were designed. Primer_I and Primer_D were used in the I reaction, which yielded an I-type chromosome-specific 3-kb PCR product, while Primer_A and Primer_D were used in the A reaction, which was A-type chromosome-specific. Primer_A and Primer_E were used in the Ai reaction, which gave an I-type-specific product. A total of 10 ng genomic DNA was used in each reaction. I- or A- type was judged after 20 cycles of amplification with PrimeSTAR Max DNA polymerase (Takara Bio Inc., Shiga, Japan).

For Southern blotting, *H. polymorpha* genomic DNA was prepared using a standard protocol [46]. Briefly, 3 µg DNA was digested with *Eco*RI, *Xho*I, *Pst*I, and *Bam*HI restriction enzymes before electrophoresis. A standard protocol was used for blotting and hybridization [46]. DNA probes were prepared and detection was performed using the AlkPhos Direct Labeling and Detection System with CDP-Star (GE Healthcare, Pittsburgh, PA, USA).

### Semi-quantitative mating

Yeast strains of *leu1-1* or *ura3-1* genotypes were grown at 30°C in YPDS until the optical density at 663 nm (A663) was between 0.5 and 1.5. Cells were washed with PBS and diluted to A663 = 1.0, and a 10-µl cell suspension of the two strains was mixed on a nitrocellulose membrane filter that was placed on a MEMA plate and incubated for 24 h at 30°C. Cells were re-suspended in PBS and dilutions were plated on SD plates supplemented with leucine or uracil or on unsupplemented SD plates that were incubated for 2 days at 37°C. The mating percentage was calculated as the number of colonies on unsupplemented plates divided by the number on leucine- or uracil-supplemented plates (i.e., whichever had fewer colonies). It should be noted that the mating percentage does not represent overall mating efficiency because meiosis and sporulation proceed immediately after mating in *H. polymorpha*.

### RNA analysis

Total RNA was isolated from *H. polymorpha* as previously described [47], treated with DNase I, and then further purified using the RNeasy Plus Kit (Qiagen, Valencia, CA, USA). A total of 1 µg RNA was used to synthesize cDNA with SuperScriptIII (Invitrogen, Carlsbad, CA, USA) according to the manufacturer's protocol, and 1 µl cDNA reaction mixture was used in a PCR reaction with the primers listed in Table S2. The QuantStudio 3D Digital PCR system (Thermo Fisher Scientific Inc., Waltham, MA, USA) was used to quantify RNA copy number. Forward, reverse, and TaqMan primers are listed in Table S2.

## Supporting Information

Figure S1 Gene organization of the MAT locus in yeast species. *MAT* loci are marked by yellow. The organization of the α idiomorph is shown in the upper line and that of the **a** idiomorphs in the lower line for each species. ORFs are shown as thick arrows. Arrows are not drawn to scale. Coloured arrows indicate homologous genes: red, *α1*; pink, *α2*; blue, **a** *1*; dark blue, **a** *2*; purple, *SLA2*; green, *SUI1*; light purple, *DIC1*; light blue, *BUD5*; light green, *PHO87*. Phyrogenic relationship is shown on the left. The tree is not drawn to scale.
(TIF)

Figure S2 DNA sequences of *MAT1* locus. Predicted amino acid sequences of α2, α1, and **a**1 were indicated in pink, red, and blue, respectively. Drawn by SnapGene version 2.4.2 (GSL Biotech LLC, Chicago, IL, USA).
(TIF)

Figure S3 Amino acid sequence alignment of **a**1 proteins from yeast species. Amino acid sequences of a1 proteins in *Candida glabrata* (Cg_a1), *Debaryomyces hansenii* (Dh_a1), *K. lactis* (Kl_a1), *S. cerevisiae* (Sc_a1), *Pichia pastoris* (Pp_a1), *Zygosaccharomyces rouxii* (Zr_a1), *Ashbya gossypii* (Ag_a1), *O. parapolymorpha* (Op_a1) as well as *H. polymorpha* **a**1 (Hp_a1) and **a**1* (Hp_a1s) were aligned by Clustal Omega (1.2.1) (Text S1).
(TIF)

Figure S4 Homothallic mating in cells deleted for pheromone receptors. (A) Schematics of the experiment in Figure 3C. Mating in crosses between wild type (i), wild type and *Ste2*Δ (ii), and wild type and *ste3*Δ (iii) were shown. Presence of **a** and α mating pheromones as well as their receptors were presumed based on the *S. cerevisiae* mating system. (B) Schematics of the experiment in Figure 3D. Cells with α cell identity are presumed absent in *α1*Δ cells based on the result in Figure 3B.
(TIF)

Figure S5 Mating assay of *α1*-expressing strains with *Ste2*Δ and *ste3*Δ strains. *α1* expression from the *TEF1* promoter negatively affects mating efficiency with *Ste2*Δ but not with *ste3*Δ. Cells were treated as described in Fig. 2A. Shown is the average of three independent matings. Error bars indicate SD.
(TIF)

Figure S6 The inversion was detected at low frequency in mitotically growing cells. (A) Inverted type was detected in PCR using primers within the MAT intervening region. Genomic DNA was prepared from HPH22 cells and PCR reactions were carried out with Primer_7, 9, 14, or 16 together with Primer_M. Primers_7, 9 and Primers_14, 16 anneal to the opposite DNA strand. After 30 cycles of amplification, specific PCR products were present in reactions with Primer_7 as well as that with Primer_14 and Primer_16. (B) Schematic drawing of isolation of HPH22 and HPH22i strain. A- or I-type was determined by PCR.
(TIF)

Figure S7 RT-PCR analysis of *α1* and **a** *1* genes. RNA samples were prepared from logarithmically growing A-type wild type cells (HPH22i). Primer MAT-7 and Primer MAT-8 were used for PCR to detect *α1*-**a** 1* cDNA. M: 1 kb DNA ladder (New England BioLabs, Inc., Ipswich, MA, USA).
(TIF)

Figure S8 *IR_2_*Δ cells are defective for the inversion. PCR reactions in Fig. 5B were amplified 25 cycles. Ai product was not detected in *IR_2_*Δ genomic DNA.
(TIF)

Figure S9 Inversion does not alter the expression of *FAS2* gene. RT-PCR analysis of *FAS2* gene. RNA samples were prepared from I- (HPH22) or A- (HPH22i) type wild-type cells incubated on MEMA medium for 15 hrs. Primers used for PCR are listed in Table S2.
(TIF)

Figure S10 RT-PCR analysis of *STE2*, *STE3*, and *STE12* genes. RNA samples are the same as in Fig. 5B. Primers used for PCR are listed in Table S2.
(TIF)

Table S1 Yeast strains and plasmids.
(XLSX)

Table S2 Primer sequences.
(XLSX)

Text S1 Supplemental reference.
(DOCX)
