# Supplementary figures and images for: Inversion of the Chromosomal Region between Two Mating Type Loci Switches the Mating Type in Hansenula polymorpha
Source: PLoS Genet. 2014 Nov 20;10(11):e1004796. doi: 10.1371/journal.pgen.1004796 (PMC4238957; doi:10.1371/journal.pgen.1004796)

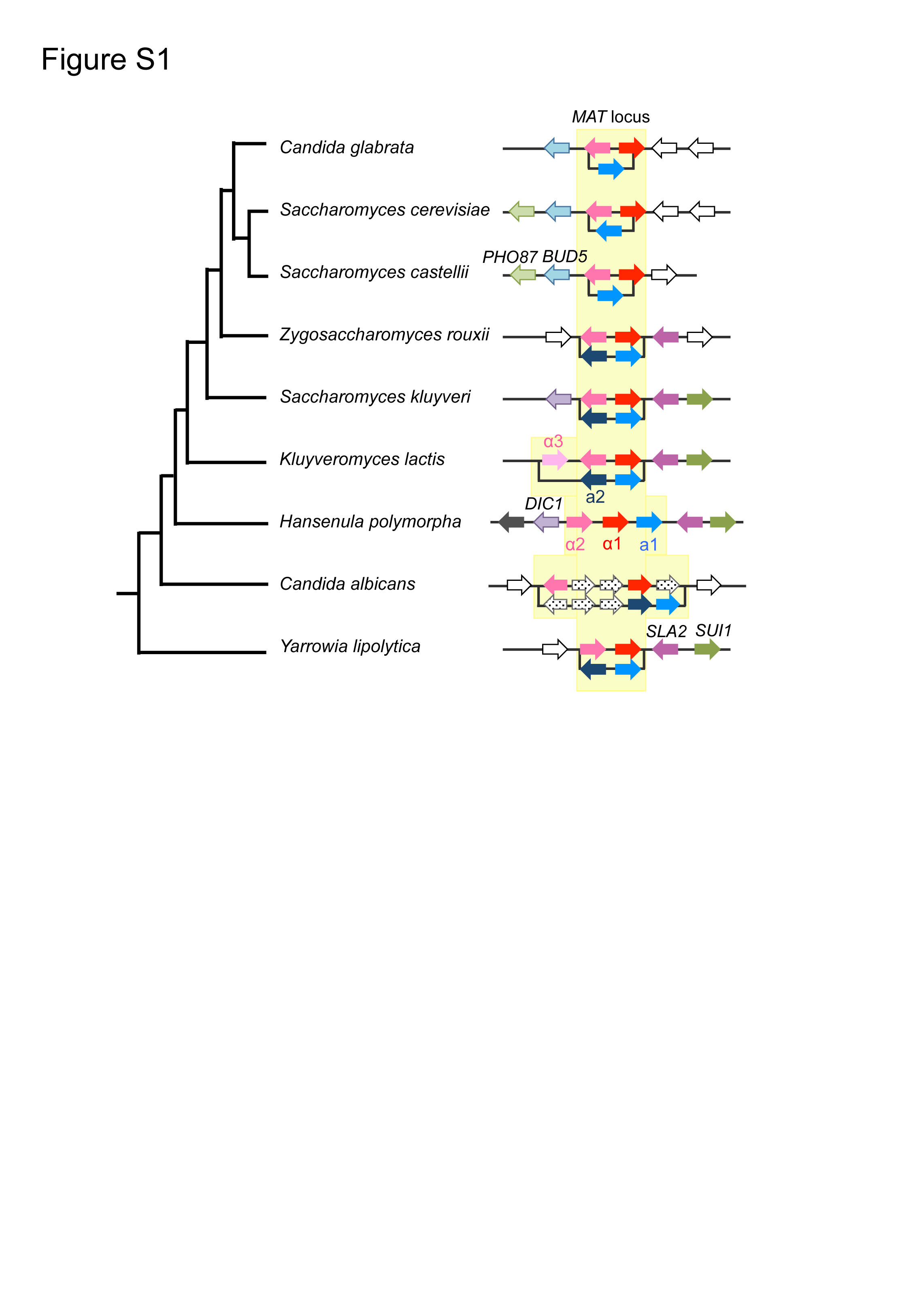

Supplement: Figure S1 — Gene organization of the MAT locus in yeast species. MAT loci are marked by yellow. The organization of the α idiomorph is shown in the upper line and that of the a idiomorphs in the lower line for each species. ORFs are shown as thick arrows. Arrows are not drawn to scale. Coloured arrows indicate homologous genes: red, α1; pink, α2; blue, a 1; dark blue, a 2; purple, SLA2; green, SUI1; light purple, DIC1; light blue, BUD5; light green, PHO87. Phyrogenic relationship is shown on the left. The tree is not drawn to scale. (TIF) [file pgen.1004796.s001.tif]

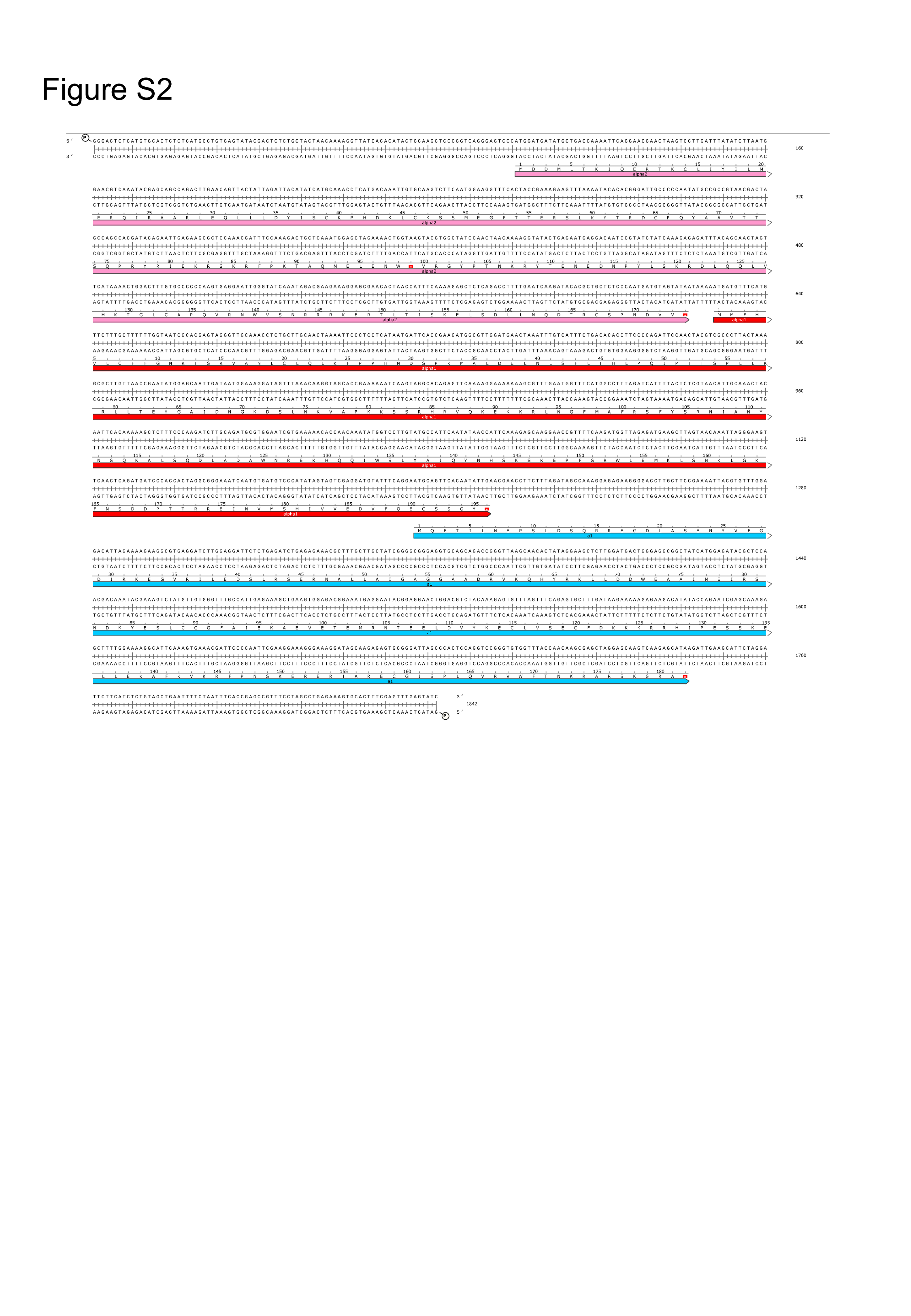

Supplement: Figure S2 — DNA sequences of MAT1 locus. Predicted amino acid sequences of α2, α1, and a1 were indicated in pink, red, and blue, respectively. Drawn by SnapGene version 2.4.2 (GSL Biotech LLC, Chicago, IL, USA). (TIF) [file pgen.1004796.s002.tif]

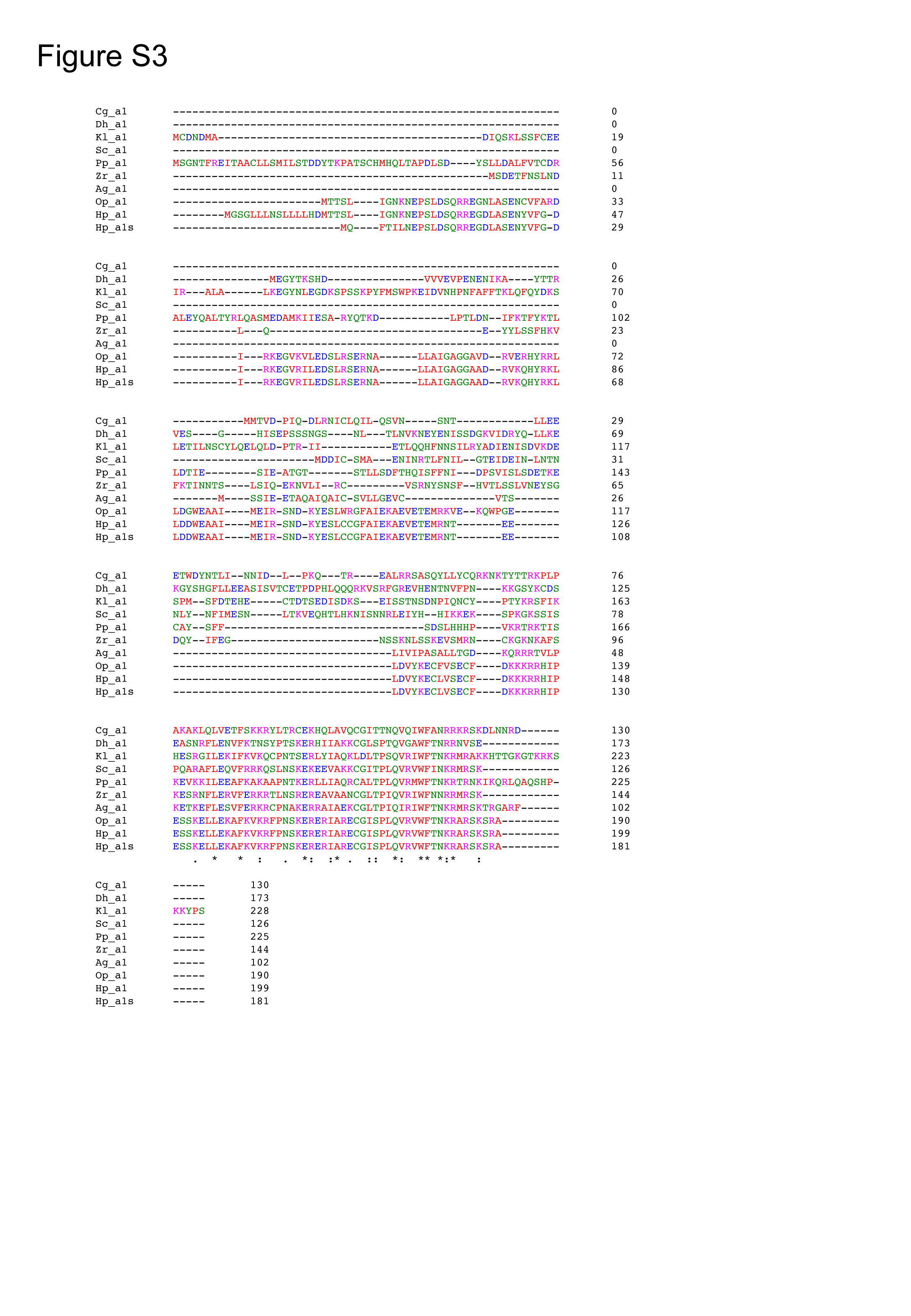

Supplement: Figure S3 — Amino acid sequence alignment of a1 proteins from yeast species. Amino acid sequences of a1 proteins in Candida glabrata (Cg_a1), Debaryomyces hansenii (Dh_a1), K. lactis (Kl_a1), S. cerevisiae (Sc_a1), Pichia pastoris (Pp_a1), Zygosaccharomyces rouxii (Zr_a1), Ashbya gossypii (Ag_a1), O. parapolymorpha (Op_a1) as well as H. polymorpha a1 (Hp_a1) and a1* (Hp_a1s) were aligned by Clustal Omega (1.2.1) (Text S1). (TIF) [file pgen.1004796.s003.tif]

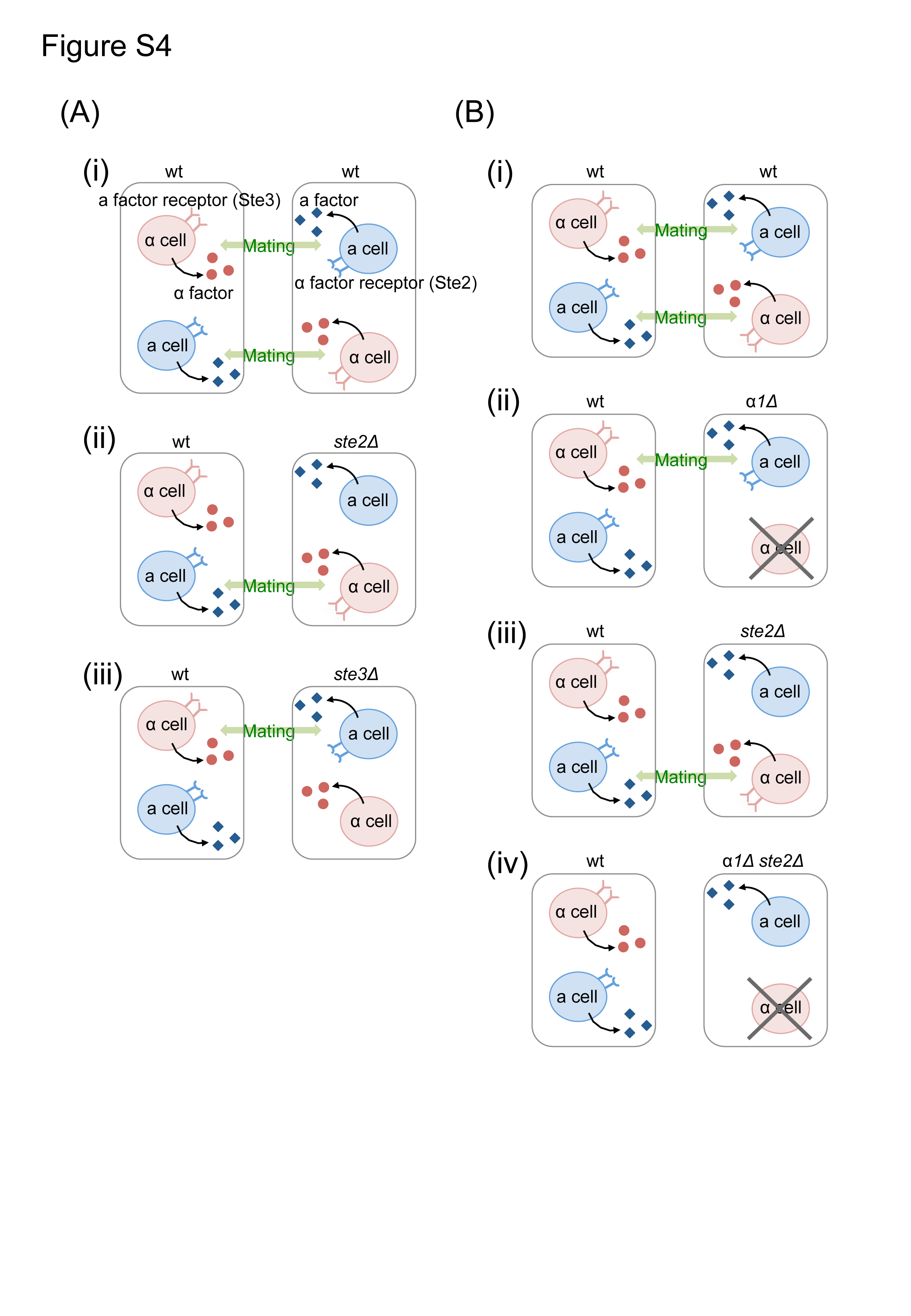

Supplement: Figure S4 — Homothallic mating in cells deleted for pheromone receptors. (A) Schematics of the experiment in Figure 3C. Mating in crosses between wild type (i), wild type and Ste2Δ (ii), and wild type and ste3Δ (iii) were shown. Presence of a and α mating pheromones as well as their receptors were presumed based on the S. cerevisiae mating system. (B) Schematics of the experiment in Figure 3D. Cells with α cell identity are presumed absent in α1Δ cells based on the result in Figure 3B. (TIF) [file pgen.1004796.s004.tif]

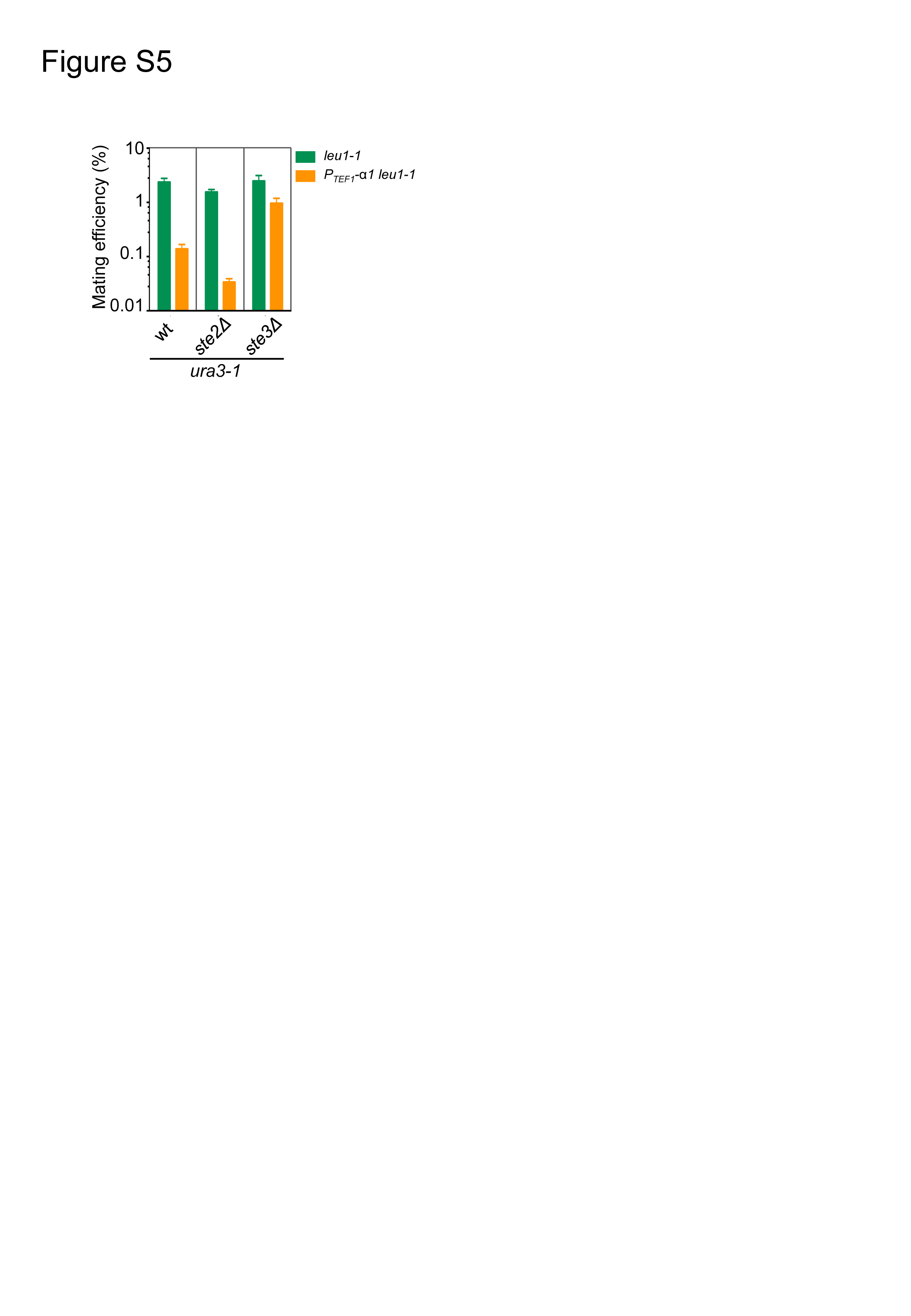

Supplement: Figure S5 — Mating assay of α1-expressing strains with Ste2Δ and ste3Δ strains. α1 expression from the TEF1 promoter negatively affects mating efficiency with Ste2Δ but not with ste3Δ. Cells were treated as described in Fig. 2A. Shown is the average of three independent matings. Error bars indicate SD. (TIF) [file pgen.1004796.s005.tif]

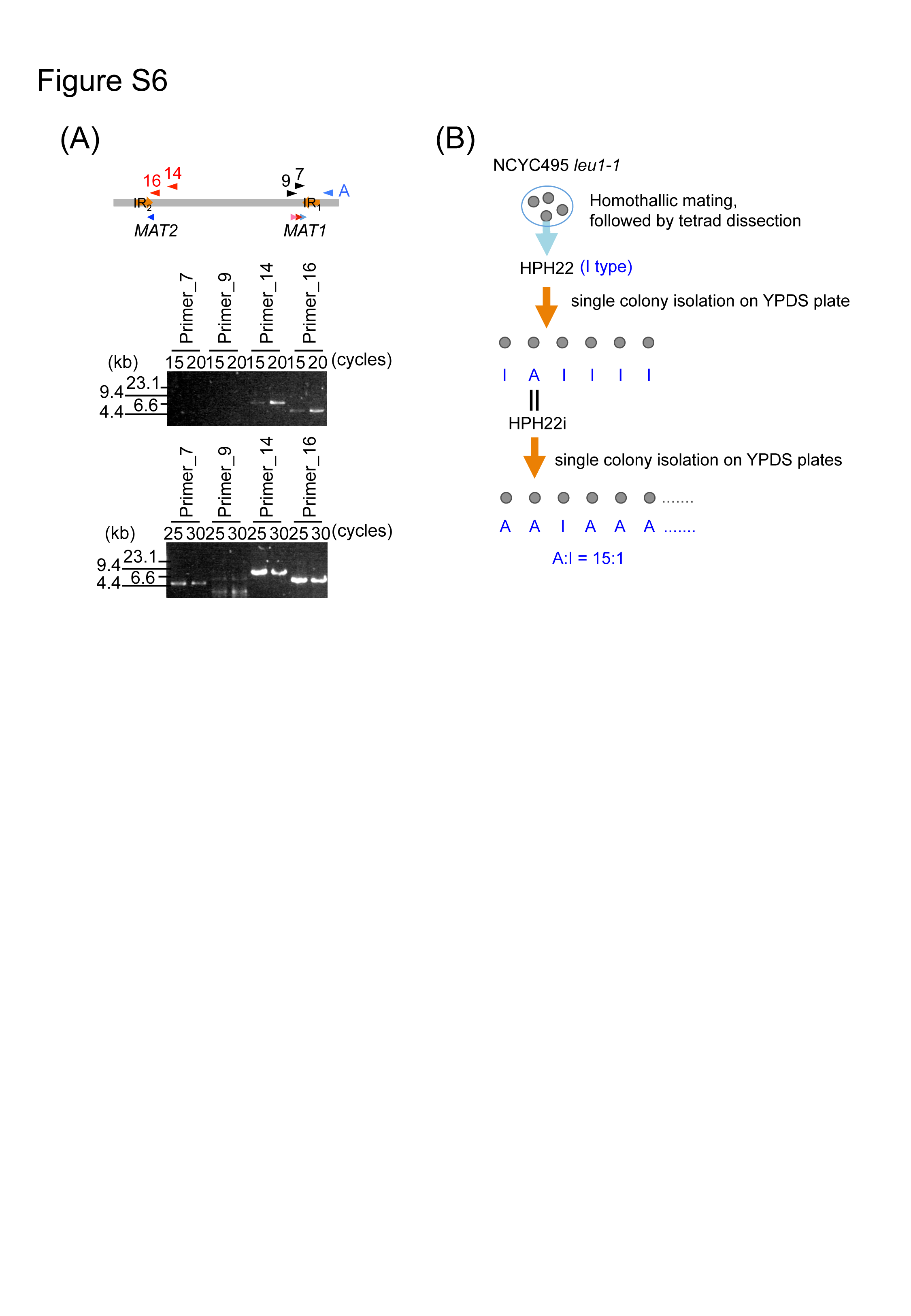

Supplement: Figure S6 — The inversion was detected at low frequency in mitotically growing cells. (A) Inverted type was detected in PCR using primers within the MAT intervening region. Genomic DNA was prepared from HPH22 cells and PCR reactions were carried out with Primer_7, 9, 14, or 16 together with Primer_M. Primers_7, 9 and Primers_14, 16 anneal to the opposite DNA strand. After 30 cycles of amplification, specific PCR products were present in reactions with Primer_7 as well as that with Primer_14 and Primer_16. (B) Schematic drawing of isolation of HPH22 and HPH22i strain. A- or I-type was determined by PCR. (TIF) [file pgen.1004796.s006.tif]

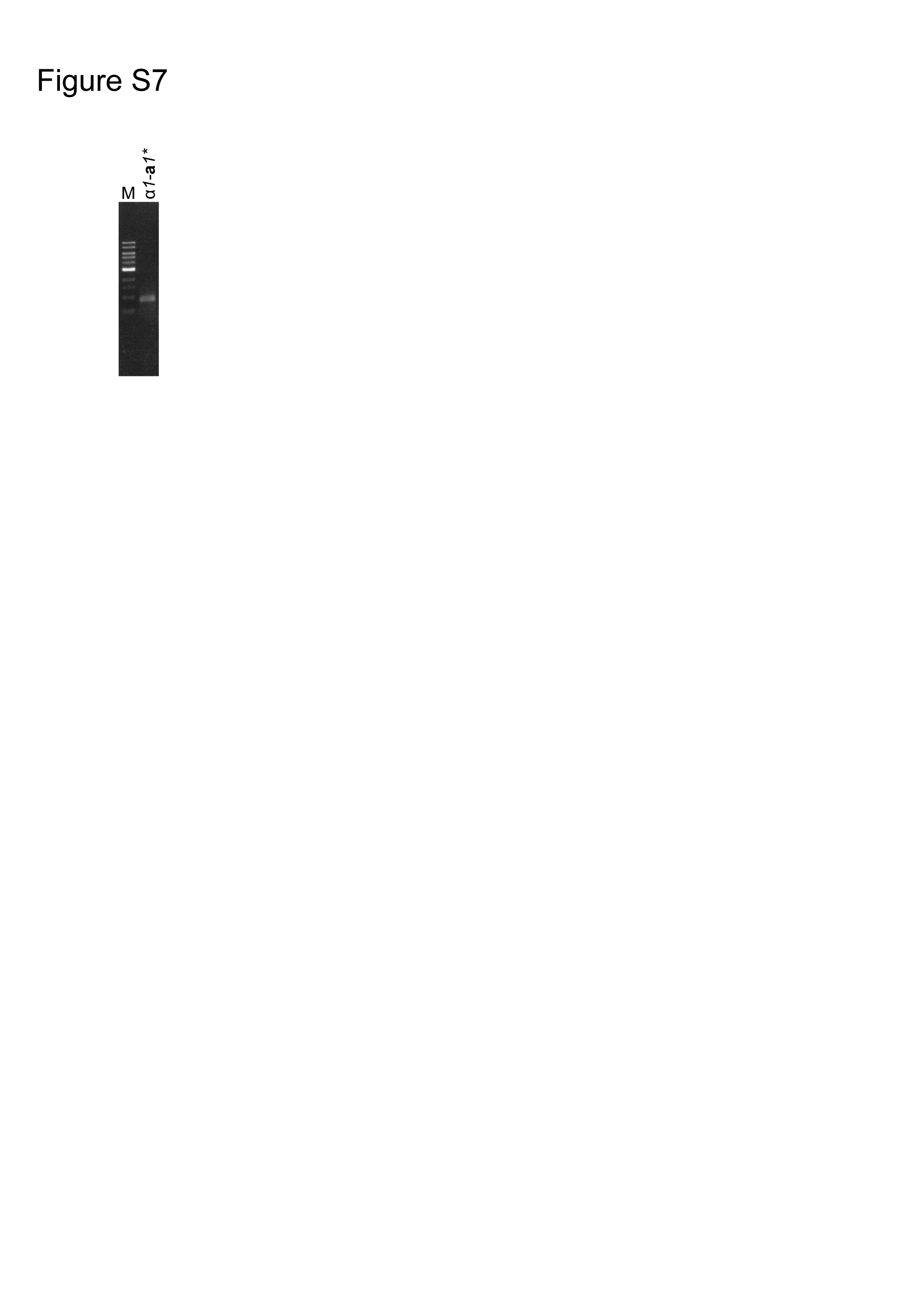

Supplement: Figure S7 — RT-PCR analysis of α1 and a 1 genes. RNA samples were prepared from logarithmically growing A-type wild type cells (HPH22i). Primer MAT-7 and Primer MAT-8 were used for PCR to detect α1-a 1* cDNA. M: 1 kb DNA ladder (New England BioLabs, Inc., Ipswich, MA, USA). (TIF) [file pgen.1004796.s007.tif]

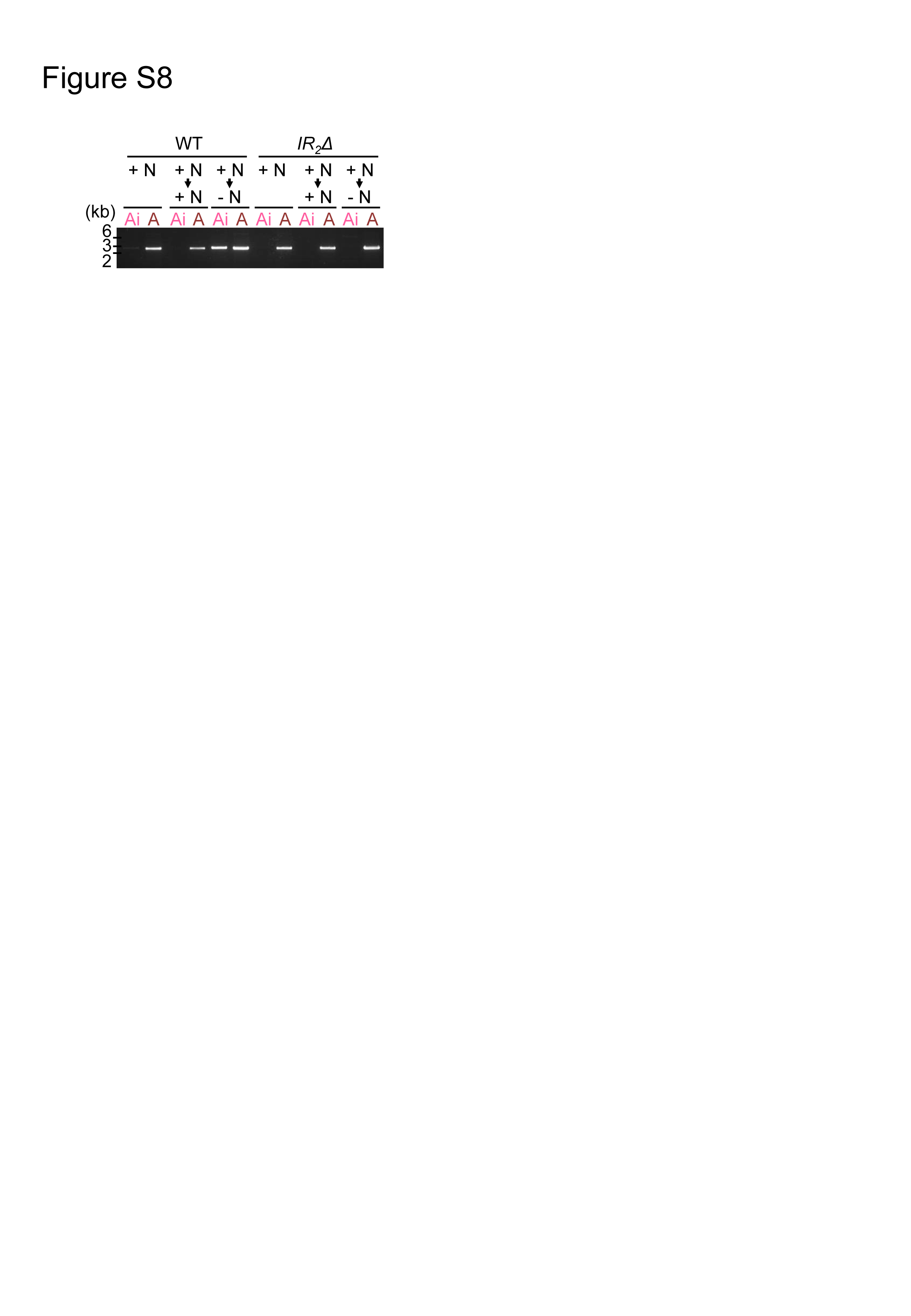

Supplement: Figure S8 — IR2Δ cells are defective for the inversion. PCR reactions in Fig. 5B were amplified 25 cycles. Ai product was not detected in IR2Δ genomic DNA. (TIF) [file pgen.1004796.s008.tif]

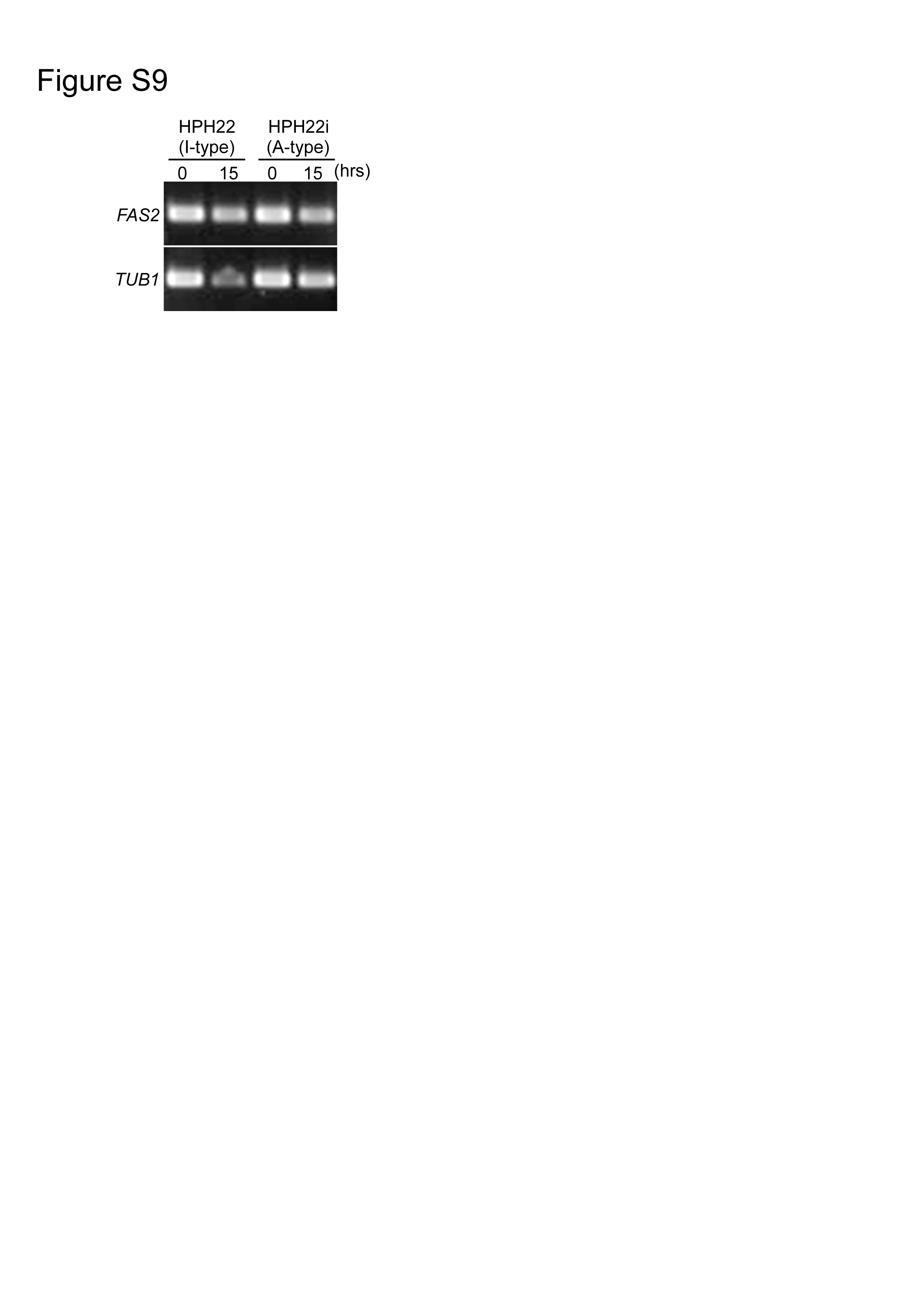

Supplement: Figure S9 — Inversion does not alter the expression of FAS2 gene. RT-PCR analysis of FAS2 gene. RNA samples were prepared from I- (HPH22) or A- (HPH22i) type wild-type cells incubated on MEMA medium for 15 hrs. Primers used for PCR are listed in Table S2. (TIF) [file pgen.1004796.s009.tif]

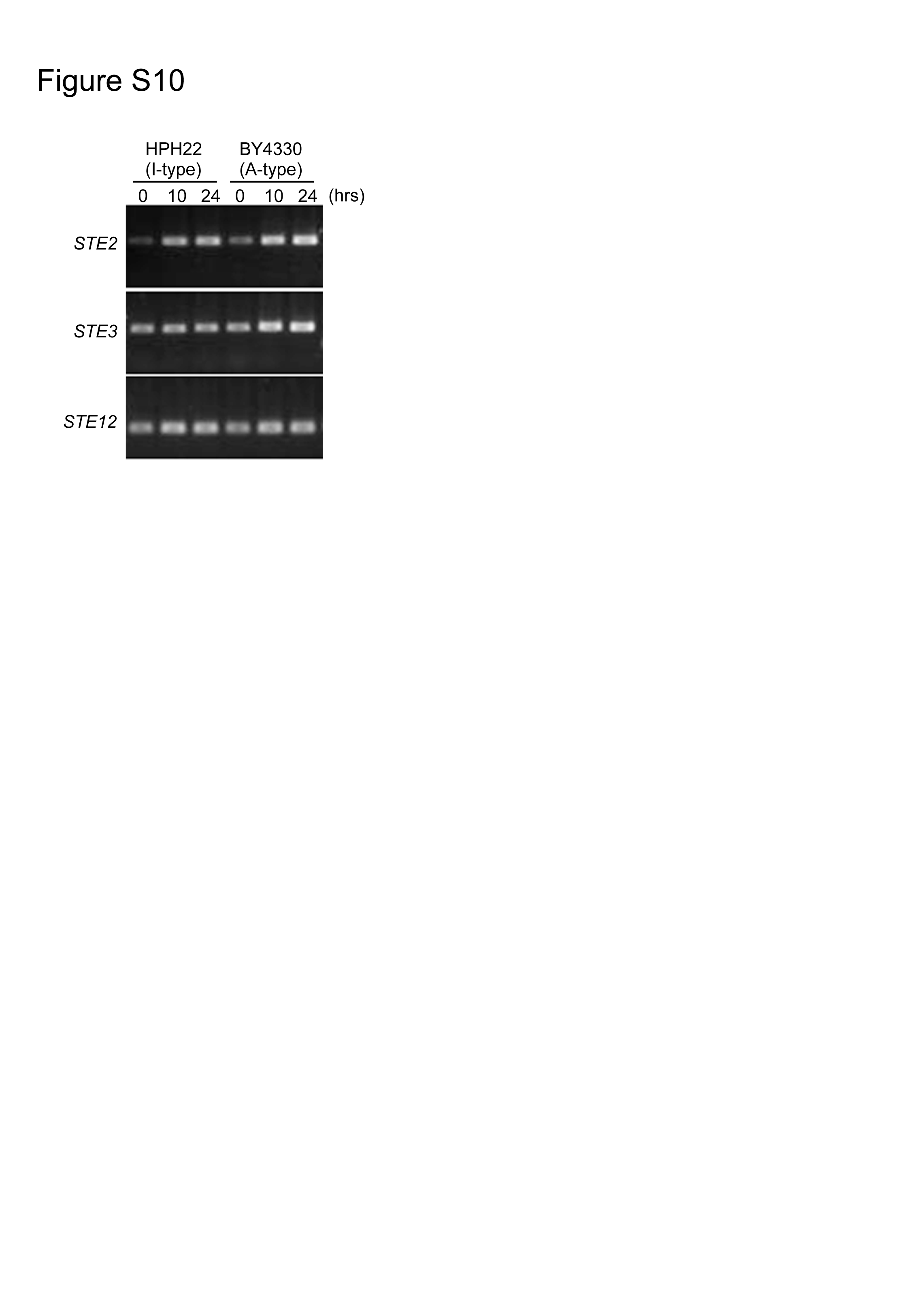

Supplement: Figure S10 — RT-PCR analysis of STE2, STE3, and STE12 genes. RNA samples are the same as in Fig. 5B. Primers used for PCR are listed in Table S2. (TIF) [file pgen.1004796.s010.tif]
