# Supplementary material for: Inversion of the Chromosomal Region between Two Mating Type Loci Switches the Mating Type in Hansenula polymorpha
Source: PLoS Genet. 2014 Nov 20;10(11):e1004796. doi: 10.1371/journal.pgen.1004796 (PMC4238957; doi:10.1371/journal.pgen.1004796)
Supplement: Text S1 — Supplemental reference. (DOCX) [file pgen.1004796.s013.docx]

Text S1 Supplemental reference

S1. Sievers F, Wilm A, Dineen D, Gibson TJ, Karplus K, et al. (2011) Fast, scalable generation of high-quality protein multiple sequence alignments using Clustal Omega. Molecular Systems Biology 7: 1–6. doi:10.1038/msb.2011.75.

S2. Goujon M, McWilliam H, Li W, Valentin F, Squizzato S, et al. (2010) A new bioinformatics analysis tools framework at EMBL-EBI. Nucleic Acids Res 38: W695–W699. doi:10.1093/nar/gkq313.
